# Sealing of calcified plaques after bioresorbable scaffold implantations: a five-year follow up

**DOI:** 10.1007/s10554-016-1035-5

**Published:** 2016-12-21

**Authors:** Erhan Tenekecioglu, Christos V. Bourantas, Yoshinobu Onuma, Patrick W. Serruys

**Affiliations:** 1 Department of Interventional Cardiology, Erasmus University Medical Center, Rotterdam, The Netherlands; 2 Department of Cardiolovascular Sciences, University College of London, London, UK; 3 Department of Cardiology, Barts Health NHS Trust, London, UK; 4 Imperial College, London, UK; 5 Emeritus Professor of Medicine Erasmus University, Rotterdam, The Netherlands

Optical coherence tomography (OCT) has been the imaging technique for detection of scaffold malapposition during implantation, the method of choice at follow up for assessing strut resorption and measurement of cap thickness overlying atherosclerotic plaques [1]. Bioresorbable scaffold (BRS) has the capability of sealing plaque by creating a neointimal cap [2]. Herewith, we present three cases from Absorb Cohort-B trial exhibiting the evolution of neointimal tissue overlying calcified plaques at 5-year post-implantation. The scaffolds were assessed on OCT after implantation, at 1-, 3- and 5-year follow ups.

**Fig. 1:**
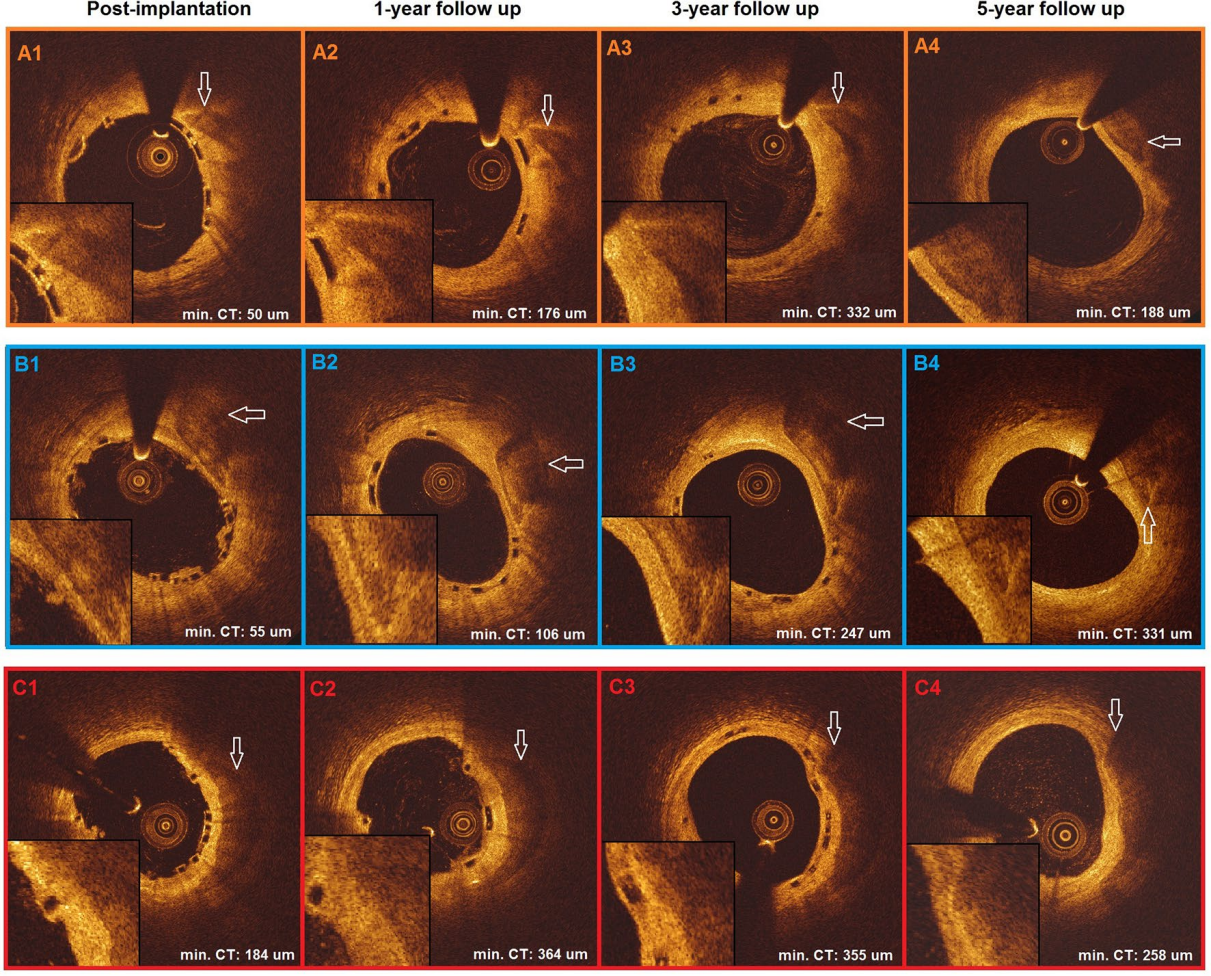
Three cases from Absorb Cohort-B trial demonstrate calcified plaques covered by scaffold struts after post implantation (*A1, B1, C1*) in optical coherence tomography images. At 1-year (*A2, B2, C2*) and 3-year follow-ups (*A3, B3, C3*), scaffold struts and progress of neointima at 1-year and at 3-years, sealing the calcified plaques, could be observed. At 5-year follow up (*A4, B4, C4*), after resorption of the struts has been completed, the calcified plaques are definitely sealed by the overlying neointimal tissue that has a tendency to shrink and reflect more intensively (*min CT* minimum cap thickness)

Coronary artery calcification is a risk factor for coronary adverse events [3]. Despite lower radial strength as compared to the metallic equivalent, Absorb is capable to stretch the non-calcified sectorial segment of the vessel lumen while creating a neointimal cap overlying the calcified plaques thereby refurbishing the endoluminal lining at that specific site (Fig. 1). Through its sealing function, Absorb further isolates the calcified plaques from the lumen and the flowing blood.

Bioresorbable scaffolds may be a treatment option for calcified plaques by inducing a neointimal cap at follow up. The advantage of biodegradable device compared to the metallic stent is temporary vessel scaffolding which reduces the inflammatory response in treated segments at follow up. Reduced inflammation with re-capping may decrease the vulnerability risk of the plaques.
